# Fishing community resilience to harmful algal blooms: social capital narratives of the 2016 crisis in southern Chile

**DOI:** 10.3389/fpubh.2026.1872462

**Published:** 2026-08-05

**Authors:** Andrés Marín, Patricio A. Díaz, Diego Valdivieso

**Affiliations:** 1 Departamento de Ciencias Sociales, Universidad de Los Lagos, Osorno, Chile; 2 Universidad de Los Lagos Centro i-mar, Puerto Montt, Chile; 3 Administración Pública, Escuela de Gobierno, Facultad de Economía, Negocios y Gobierno, Universidad San Sebastián, Sede Pichipelluco, Puerto Montt, Chile

**Keywords:** adaptive capacity, environmental governance, crisis communication, local ecological knowledge, participatory research, red tide, small-scale fisheries

## Abstract

Harmful algal blooms (HABs) represent a critical environmental challenge for coastal social-ecological systems, generating risks to human health, livelihoods, food security, and ecosystem services. The unprecedented 2016 HAB crisis in southern Chile, triggered by the northward expansion of the paralytic toxin-producing dinoflagellate *Alexandrium catenella*, caused extended fishing closures and severe socio-economic disruptions across artisanal fishing communities in the Los Lagos and Los Ríos regions. Despite effective monitoring and management systems that largely prevented human poisoning, previous research has highlighted persistent challenges related to trust, participation, communication, and the integration of local knowledge in HAB governance. To examine these social and relational dimensions, this study analyzes community narratives of the 2016 HAB crisis through a social capital lens, drawing on 64 participants engaged in five participatory workshops conducted in the rural coastal villages of Mississippi, Huiro, Bahía Mansa, Pasaje Amortajado, and Carelmapu. Using a participatory elicited letter-writing approach, we collected and analyzed narratives associated with bonding, bridging, and linking social relationships to: (1) document how participants represented the impacts and consequences of the HAB crisis; (2) examine how social relationships were described and attributed meaning in participants’ accounts; and (3) explore the implications of these narratives for understanding adaptive capacity, community resilience, and future HAB governance. The results portray HABs as protracted socio-environmental crises marked by economic disruption, emotional distress, dietary changes, and interpretations of environmental change. Bonding narratives emphasized family support, intergenerational knowledge, and cultural continuity. Bridging narratives highlighted the circulation of experiential warnings, environmental observations, and calls for collective organization among fishing communities. Linking narratives expressed concerns regarding institutional transparency, communication, and participation in environmental governance. Across all three dimensions, the letters transformed memories of the 2016 crisis into future-oriented reflections, communicating lessons, concerns, warnings, and recommendations directed toward future generations and future HAB events. The findings demonstrate the value of interpreting community narratives through a social capital lens for understanding how coastal communities experience, remember, and interpret environmental crises. By revealing the social resources, collective memories, and future-oriented reflections embedded in local narratives, this study contributes to discussions of adaptive capacity and community resilience while highlighting the potential of participatory narrative approaches to inform more legitimate, participatory, and adaptive HAB governance.

## 1 Introduction

Harmful algal blooms (HABs or “red tides”^1^) represent an escalating global environmental challenge, increasingly impacting coupled human–environment systems through toxins that threaten human health, maritime livelihoods, and food security (1, 2). While HAB occurrences have often been perceived as increasing in frequency, intensity, and geographic extent over the past decades (3), global syntheses (4) have found no uniform global trend once differences in monitoring effort are taken into account, with some regions showing increases and others stable or declining patterns (5). In this sense, the apparent worldwide increase in HABs largely reflects intensified monitoring and the emergence of high-impact events, rather than a simple global rise in all bloom types (6). At the same time, global assessments underscore that HABs continue to pose substantial and, in many places, growing risks to coastal communities, aquaculture, fisheries, and food security—from the Gulf of Mexico and New England to New Zealand and coastal Europe—and that their variability and impacts must be understood at regional and species-specific scales (4). These events, classified as biological hazards that can introduce chemical toxins into marine food webs, are driven by a complex interplay of anthropogenic nutrient enrichment and climate-driven anomalies (7–9).

Biologically, HAB can manifest as either abrupt ecosystem shocks or more incremental and creeping environmental changes. Mostly, these blooms represent longstanding and cyclical phenomena with recurring and predictable impacts on ecosystems, public health and local economies (5, 10). In other cases, toxic microalgal blooms spread to new areas, causing surprise and unprecedented consequences (11, 12). In general, HAB events often trigger protracted, slow-onset crises that evolve into long-term socio-ecological disasters. These “slow disasters” exacerbate systemic vulnerabilities that accumulate over time, ultimately testing the resilience and adaptive capacity of coastal fishing communities (13, 14).

Southern Chile is recognized as a global hotspot for deeply disruptive blooms, as dramatically illustrated during the unprecedented 2016 crisis (8, 15). During this event, a massive bloom of paralytic toxin-producer dinoflagellate *Alexandrium catenella* affected the Chiloé Archipelago and expanded ca. 235 km north of the northern boundary known until 2009 (16). The 2016 bloom caused fishing closures for extended periods in several localities (17, 18). This regime shift triggered severe economic shocks and widespread social unrest in a vast geographical zone (19), exposing the vulnerabilities of a large population in general, and of benthic-resource dependent communities in particular (20). Several studies have reported broad impacts on various stakeholder groups, particularly in the Chiloé archipelago (21), but a significant gap remains in exploring the social and cultural aspects of the disaster among small-scale fishers and their communities at the local level.

The governance and management of HABs in Chile involve a multi-institutional framework coordinated primarily by the State (18, 22). The Ministry of Health (MINSAL), through its Regional Ministerial Secretariats (SEREMIs) and the Public Health Institute (ISP), manages the national surveillance program to prevent human intoxications and safeguard public health. In the framework of this program, toxicity in shellfish (including *C. concholepas*) is analyzed by mouse bioassay (MBA) following the Association of Official Analytical Chemists (AOAC) method 959.08 (23). Technical monitoring of toxic species and marine toxins is primarily conducted by the Fisheries Development Institute (IFOP^2^), which maintains systematic monitoring programs across the southern fjord systems and the Pacific coast under the direction of the Undersecretariat of Fisheries and Aquaculture (SUBPESCA). The National Fisheries and Aquaculture Service (SERNAPESCA) complements these efforts through the Shellfish Sanitation Program (PSMB), which ensures the safety of benthic resources for both domestic consumption and international export. Furthermore, several university programs and research centers—such as the Cimar-Fiordos oceanographic program and other university-based laboratories—provide scientific grounds for these efforts, generating long-term environmental baselines and fostering transdisciplinary knowledge transfer to coastal communities (18).

While Chile has achieved high levels of technical monitoring and institutional effectiveness that have substantially reduced human fatalities over recent decades (22, 24), the system frequently lacks social legitimacy and trust among fishing communities (25, 26). Previous assessments have also characterized HAB governance in Chile as predominantly reactive, relying on fragmented management efforts that hinder more comprehensive and anticipatory approaches to addressing the problem (18). Beyond their technical dimensions, effective HAB monitoring and management depend heavily on social processes. Previous research has shown that risk communication, public trust, stakeholder participation, local ecological knowledge, and collaboration between institutions and resource users are critical factors influencing the legitimacy, acceptance, and effectiveness of HAB monitoring, mitigation, and adaptation measures (22, 27). In coastal resource-dependent communities, responses to HAB events are shaped not only by scientific information and regulatory actions, but also by how affected actors interpret environmental risks, evaluate institutional credibility, and mobilize local knowledge and social networks. Understanding these processes is therefore essential for developing governance approaches that are not only technically effective but also socially legitimate and adaptive. The exclusion of local actors from scientific analysis and decision-making has contributed to suspicion and recurring controversies regarding the natural or industrial origins of HAB events (18, 19). Although previous studies have generated important insights into governance failures, risk communication, and socio-political conflict, less attention has been paid to how fishing communities themselves remember, interpret, and project the significance of HAB crises. Understanding these perspectives is particularly important because environmental disruptions are experienced not only through their immediate and tangible consequences, but also through the relationships among individuals, organizations, and institutions. Resource users and coastal communities’ information, trust and participation are fundamental factors of HAB monitoring, mitigation and adaptation measures and policy. Social capital (hereafter SC) provides a useful framework for examining these relational dimensions and for exploring how they are represented in community narratives of crisis, learning, and future preparedness (28, 29).

**Figure 1:**
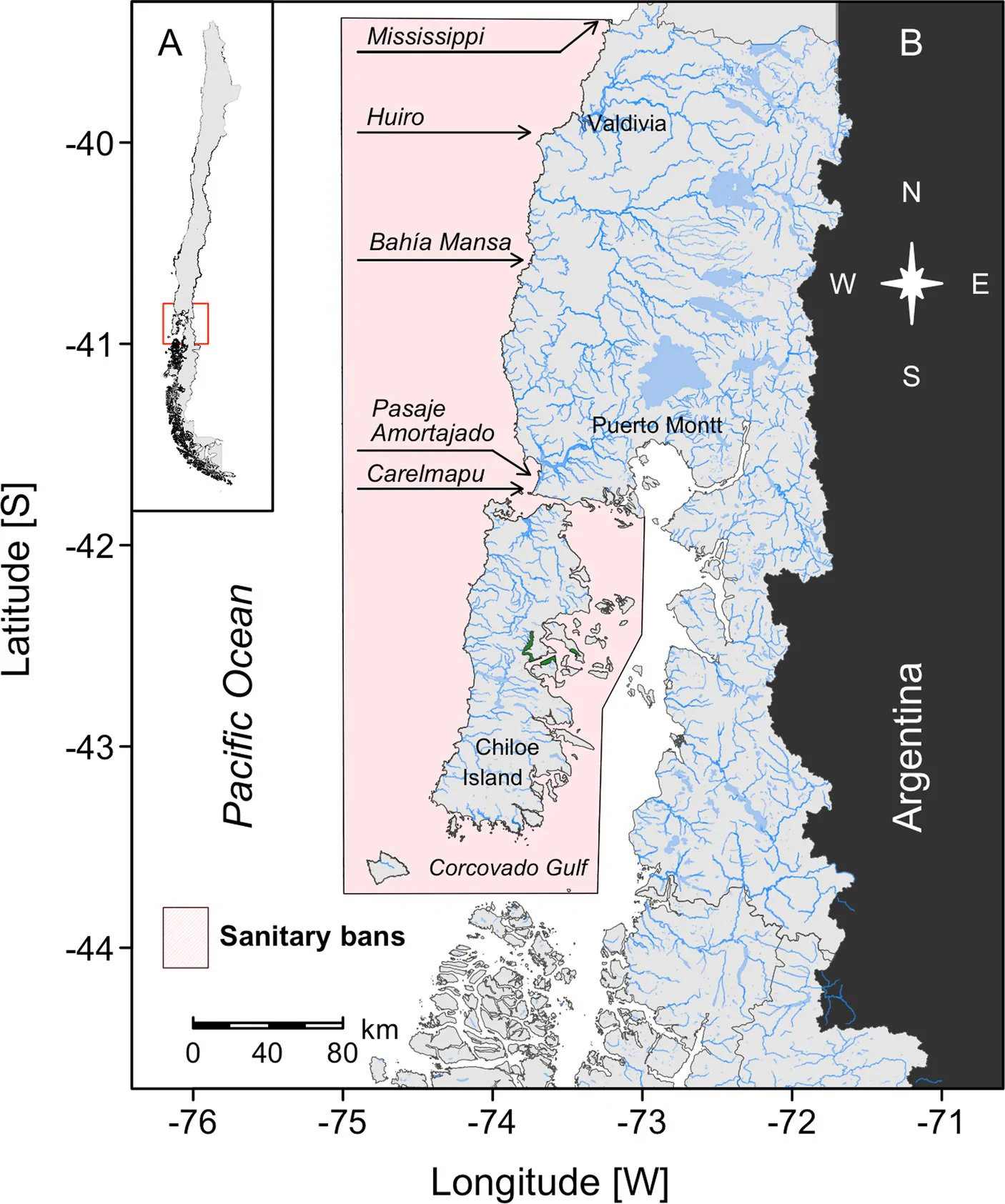
Study area in southern Chile. **(A)** Location of the study region along the Chilean coast. **(B)** Geographic extent of shellfish harvesting closures during the 2016 paralytic shellfish poisoning (PSP) outbreak and location of the five coastal villages (caletas) included in the study. The pale red polygon indicates the area affected by shellfish harvesting closures (sanitary bans), and arrows identify the study sites.

The 2016 HAB event in Chile provides a unique opportunity to draw lessons about community resilience as a social and cultural process. This study focuses on five rural coastal villages (*caletas*)—Mississippi, Huiro, Bahía Mansa, Pasaje Amortajado, and Carelmapu (Figure 1)—to characterize SC narratives through which participants reflect on the HAB crisis and envision future capacities and needs in the face of environmental disruptions. By analyzing people’s expressions and experiences, we explore sources of community resilience and vulnerability following the 2016 HAB. The three research questions (RQ) are: (1) How are the impacts and consequences of the 2016 HAB crisis represented in peoples’ narratives?; (2) How do participants narrate and attribute significance to social relationships associated with bonding, bridging, and linking SC in relation to the HAB crisis?; (3) What implications do these narratives hold for understanding and supporting community resilience and adaptive capacity, and for informing future HAB governance?

## 2 Conceptual framework

Community resilience refers to the collective capacity of local actors to take intentional action to respond to and influence the course of social and environmental change (30, 31). It represents the active articulation of multiple resources—including local ecological knowledge (LEK), community networks, and leadership—to thrive with environmental uncertainty and surprise (30). This perspective stems from the convergence of two scholarly traditions: the social-ecological systems strand, which focuses on system dynamics, feedbacks, and the capacity to absorb disturbance while retaining identity (30, 32), and the human development strand, which emphasizes identifying internal strengths and the capacity of groups to negotiate for resources in culturally meaningful ways (30, 33). The approach recognizes that resilience is based on social, cultural, and ecological dimensions, as human and environmental dynamics feed back into each other across multiple temporal and spatial scales (13, 30). Collectively, these dimensions activate community agency, transforming resilience into a situated social process through which people respond to environmental changes and crises (30, 34).

A central component of resilience is adaptive capacity, broadly understood as the ability of individuals and communities to anticipate, respond to, learn from, and adjust to environmental change and disturbance (30, 35). Adaptive capacity is shaped by multiple interacting resources and capabilities, including knowledge, leadership, institutions, and social relationships. Among these, SC has been widely recognized as an important relational dimension that can support collective action, learning, coordination, and access to resources in times of uncertainty (30, 34).

Social capital has been broadly considered a key component of community resilience. This perspective refers to an individual or group ability to act and make use of multiple resources through the existence of social relations, shared norms and mutual trust (36, 37). SC is typically operationalized at three types or levels: (1) Bonding: i.e. strong intra-community ties representing the “social glue” that sustains solidarity and identity (38); (2) Bridging: i.e., referred to as the “social lubricant,” includes horizontal ties connecting different communities and facilitating cooperation and collective action (39); (3) Linking: i.e. vertical relationships connecting communities with actors positioned within institutional hierarchies (40), which are often associated with informal insurance mechanisms and resource mobilization through “social pipelines” during crises (34, 41). The mobilization of key resources through these ties is, however, not only structural; it is simultaneously embedded in cultural patterns and depends on particular norms, memories, beliefs and knowledge systems (42, 43). Therefore, resilience constitutes a multidimensional process where actors exchange in ways that are culturally meaningful and rooted in their territorial identity (30, 33, 44).

Although SC is widely associated with trust, cooperation, and collective action, the concept has important limitations. Critics have argued that the concept may underemphasize broader structural inequalities, power relations, and institutional conditions shaping community outcomes (42). Other scholars have also warned against the potentially negative consequences of SC—the so-called “dark side”—where, for instance, strong SC among local elites reinforces existing power asymmetries and further marginalizes actors at the periphery (45, 46). In this sense, this concept should not be viewed as a “silver bullet” for addressing community challenges. Rather, it offers a framework for examining the social connections through which environmental crises are recalled. Recognizing these limitations is particularly important in crisis contexts, where social relationships and networks may simultaneously provide support while reproducing existing inequalities (42).

From the perspective of those who experience HAB, these are likely to be described not only as environmental disturbances but also as events embedded in social relationships and institutional interactions. The focus of this study is therefore not to measure SC or to assess its causal effects on resilience outcomes. Rather, this approach provides an interpretive framework for examining how participants retrospectively narrate the crisis, make sense of collective experiences, and envision capacities, needs, and aspirations for responding to environmental perturbations. A qualitative approach is particularly valuable in this regard, as it encourages participants to connect past experiences with future possibilities. The concepts of bonding, bridging, and linking SC help illuminate the relational dimensions that participants themselves emphasize when reflecting, for instance, on support, cooperation, institutional engagement during and beyond the HAB crisis.

**Figure 2:**
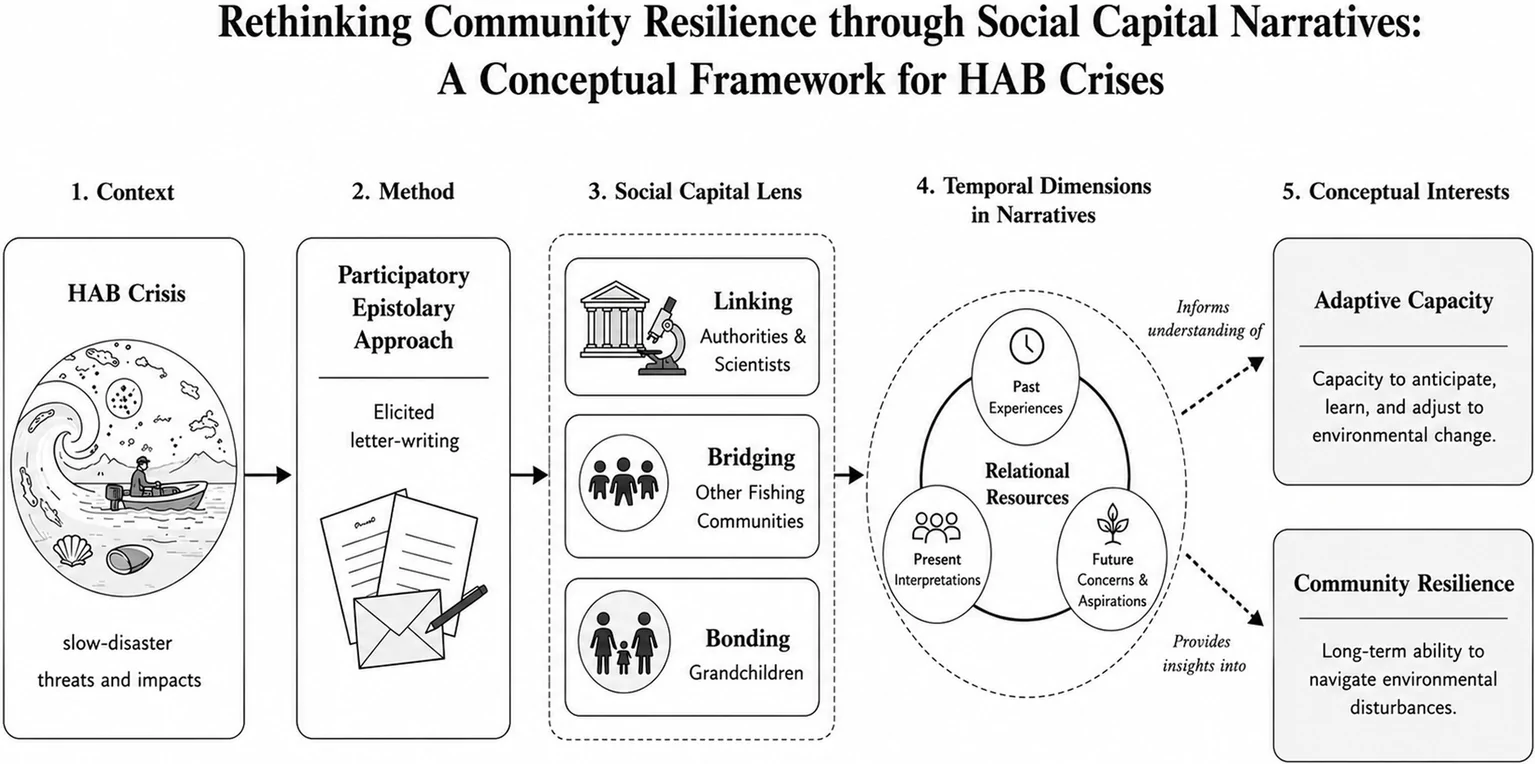
Conceptual framework guiding the study, linking a participatory epistolary approach, social capital narratives, and relational resources to broader discussions of adaptive capacity and community resilience in the context of HAB crises.

Existing HAB research has substantially advanced understanding of bloom dynamics, ecological impacts, economic losses, governance challenges, and public health consequences (27, 47). More recently, studies have also begun to examine community vulnerability, adaptive capacity, stakeholder perceptions, and social-ecological responses to HAB events (48, 49). However, comparatively less attention has been paid to how affected communities make sense of HAB crises through the social relationships, networks, and institutional connections that structure everyday life. In particular, the role of SC as a cultural and interpretive lens for understanding how HAB experiences are collectively recalled, given meaning, and projected toward future environmental disturbances remains underexplored. Methodologically, HAB studies have relied predominantly on interviews, surveys, and stakeholder-based assessments, while narrative approaches capable of simultaneously capturing retrospective reflections and future-oriented expectations remain rare. Addressing these empirical, theoretical, and methodological gaps, this study employs an epistolary elicitation approach and analyzes participants’ stories through the concepts of bonding, bridging, and linking SC. This framework helps illuminate how affected communities interpret and navigate relationships of support, cooperation, and institutional engagement during environmental crises, generating insights relevant to understanding adaptive capacity, community resilience, and future HAB governance (Figure 2).

## 3 Materials and methods

### 3.1 Research setting

HABs in Chile have followed the global trend of an “apparent” increase of toxic events due to more frequent reports and monitoring observations (6). Nevertheless, the northward expansion of *A. catenella* events in the country has been evident. After the first detection in the Magallanes straight in 1972, subsequent analyses in the 80s and early 90s revealed its presence in the Aysén region (50). By 1998, the progression of the affected area reached the south of Los Lagos region (20, 51), and in 2009 the northern boundary moved to the Chacao Channel (41°47′S 73°32′W), including the eastern coast of the Chiloé Archipelago. More recently, the expansion to the north continued. During summer-autumn 2016, an intense paralytic shellfish poisoning (hereafter PSP) outbreak reached for the first time as far north as Los Ríos, setting a new limit at 39°S latitude (8, 52).

The expansion of *A. catenella* blooms in North and South Patagonia is associated with increased threats and impacts to public health and to the artisanal fishing industry. Los Lagos houses the largest number of gleaners, divers and fishers in Chile [e.g., 37,196 users representing more than 35% of the national total; SERNAPESCA (53)]. And in the Los Ríos region, 4,164 additional artisanal fishers are registered. Considering only the 2016 HAB geographic extension zone, i.e., the study area of this research, an estimate of 15,145 fishers and their communities were affected by marine toxins (53). Key benthic species exploited in these regions, that are object of HAB contamination, include ‘loco’ (*Concholepas concholepas;* a species of high ecological and commercial value), ‘almeja’ (*Ameghinomya antiqua*), ‘cholga’ (*Aulacomya atra*), ‘choro’ (*Choromytilus chorus*), a‘piure’ (*Pyura chilensis*), and navajuela (*Ensis macha*).

HABs represent, in the first place, a public health threat risking the lives of seafood consumers. The toxins produced by microalgae are transferred through the food web, mainly through filter-feeding bivalves. These shellfish benthic species accumulate toxins, which are in the list of the most potent bioactive compounds (54), and cause illnesses such as PSP. HABs become a threat to fishing activities when toxin concentrations in shellfish reach levels unsafe for human consumption (regulatory levels, e.g., above 80 μg STX eq. 100 g-1 meat). In these cases, health and fisheries authorities must implement restriction measures, such as shellfish harvesting closures. The halt of natural shellfish banks and aquaculture exploitations jeopardize the base of coastal communities’ livelihoods, as is often the case in Chile.

**Figure 3:**
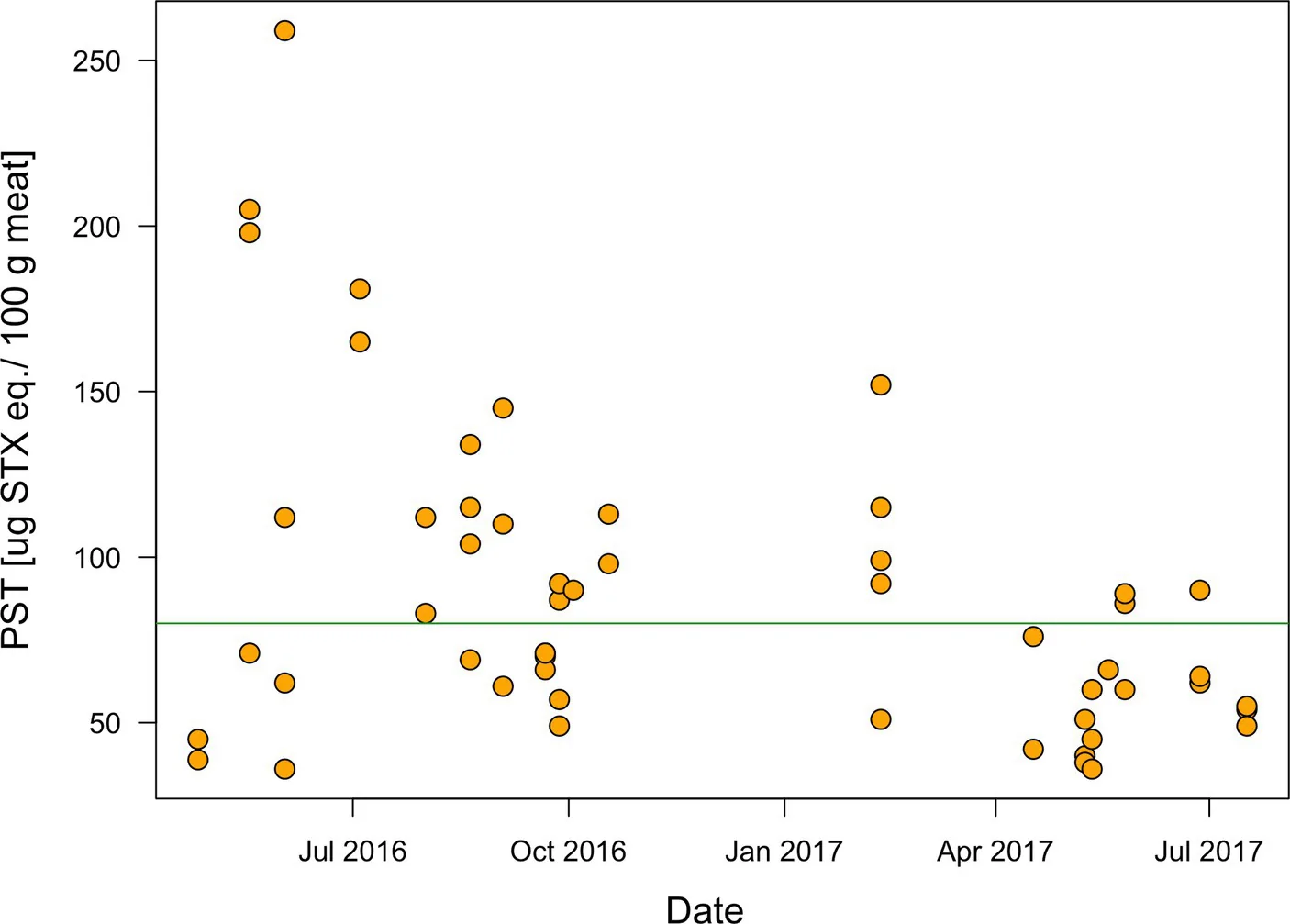
Temporal evolution of toxicity in *C. concholepas* measured by mouse bioassay in samples collected from Pasaje Amortajado between May 2016 and July 2017. The green horizontal line indicates the regulatory threshold of 80 µg STX eq. 100 g^−1^ meat. Data were obtained from the Chilean Ministry of Health Monitoring Program.

During the 2016 toxic event, most of the exposed coast of the Los Lagos and Los Ríos regions were affected by the presence of paralytic toxins above the permissible limit for human consumption. Preceded by contamination of filter-feeding organisms, contamination of ‘loco’ reached maximum concentrations of 400 μg STX. Eq. 100 g-1. Unlike filter-feeder shellfish, which exhibit a rapid detoxification phase, ‘loco’ detoxification is slow and even increases after the bloom has subsided (20). Consequently, intermittent and prolonged harvesting bans were decreed along the entire coastline of these two regions, triggering an unprecedented fisheries crisis. For instance, in Pasaje Amortajado (Los Lagos) this process exceeded 1 year, starting from maximum concentrations close to 250 μg STX eq. 100 g-1 (Figure 3). An even more extreme case was observed in Huiro (Los Ríos), where the sanitary closure lasted for more than 2 years.

Even though PSP blooms have been frequently observed in southern Chile, the risk of outbreaks in other regions is latent. Recent studies have detected toxic cells and resting cysts of *A. catenella* in the Bío-Bío region (55, 56). Understanding first-time impacts, perceptions and responses to HAB from the grassroots can shed light on coastal communities’ resilience and inform preventive and participatory policies.

### 3.2 Elicited letter writing: a participatory epistolary approach

The study employed a participatory elicited letter-writing approach, a qualitative strategy that combines insights from epistolary research, elicitation methods, and collaborative narrative production. By complementing conventional interview-based techniques, this approach invites participants to construct written narratives addressed to specific recipients, thereby revealing how experiences of crisis are articulated across different relational contexts.

Letters have long been recognized in qualitative research as valuable “documents of life,” offering insight into how individuals construct meaning, position themselves socially, and communicate moral expectations (57). As Stanley argues, letters possess three distinctive analytical features. They are dialogical, since they are written for a specific interlocutor and therefore embedded in a relational exchange. They are perspectival, because their content shifts depending on the intended recipient and historical moment. Finally, they are emergent, evolving through the unfolding interaction between writer and audience. These characteristics make letters particularly useful for examining how people narrate experiences that intertwine personal memory, collective identity, and social responsibility. As Plummer (58) notes, the analytical value of such documents lies not only in the individual accounts they preserve, but also in the collective social worlds they render visible. These worlds often become fully intelligible only in retrospect, as stories are socially shaped and “socially produced, subject to contingencies, connectedness, complexity, conflict, and contradictions” (59), p. 196. The temporal distance between the 2016 HAB crisis and the present analysis is therefore not a methodological limitation but an analytical asset: it allows the letters to be read as acts of collective memory, in which communities retrospectively give shape, meaning, and moral coherence to a shared disruption of their lives.

Building on this tradition, the present study incorporates elicitation techniques, which use prompts or stimuli to encourage participants to reflect more deeply on specific events or experiences (60). Elicitation methods are widely used in qualitative research because they help participants recall events, articulate emotions, and reflect on complex situations that may be difficult to express through direct questioning alone (61). In this study, the writing of letters itself functioned as the elicitation device. Participants were invited to write letters to different audiences, prompting them to reflect on their experience with HAB events (as described below). The epistolary tool enabled us to explore how participants experienced and interpreted their relationships of support, cooperation, communication, and institutional engagement during and after the crisis, dimensions that are increasingly recognized as important in environmental governance and adaptive responses to environmental change.

The approach also draws from traditions of collaborative and reflective letter writing in qualitative research. Collective writing has been used to explore shared experiences, emotional responses, and relational dynamics in ways that extend beyond individual accounts (62). Similarly, reflective letter-writing has been employed as a methodological tool to facilitate emotional exploration and reflexive dialogue among participants (63). By situating writing within a collaborative setting, participants can negotiate shared meanings while preserving the plurality of perspectives within the group. This process fosters co-constructed narratives that balance unity and diversity, as collaborative writing enables dialogic interplay where voices interweave without erasure (62). This emphasis on collective reflexivity aligns with broader principles of collaborative qualitative analysis, in which participants contribute actively to the interpretation of experiences and the identification of emergent themes (64).

In this study, these methodological traditions were combined into what we now formally describe as an *elicited epistolary approach*. This naming builds on a previous participatory study of volcanic risk perceptions with an indigenous community in southern Chile (65), in which the same collective letter-writing procedure was employed to elicit narratives across SC types. From that experience onward, collective letter writing has served both as a narrative data-generation technique and as a participatory space for expression and reflection on socio-ecological crises.

### 3.3 Data collection and analysis

Five participatory workshops were conducted between June 2018 and October 2021^3^ in five coastal fishing communities (or *caletas*) in southern Chile: Mississippi and Huiro in Los Ríos region, and Bahía Mansa, Pasaje Amortajado, and Carelmapu in Los Lagos region. These communities were selected because they were directly affected by, or exposed to, the geographic expansion HAB in 2016 and its associated crisis. The five study sites are situated within communes classified in the lowest tier of Chile’s Human Development Index report (66), characterized by structural socio-economic stagnation and a heavy reliance on primary-sector livelihoods. These rural territories face significant geographic isolation and severe precariousness in income and health indicators. Indigenous groups represent a substantial share of the population across the study area, ranging from 15.1% in Maullín (i.e., Carelmapu and Pasaje Amortajado) to 81.7% in San Juan de la Costa (i.e., Bahía Mansa), highlighting the relevance of indigenous territorial identity and livelihoods in the area (more detailed communal indicators are provided in Supplementary Table S1).

The interdisciplinary research team comprised male and female researchers and assistants with backgrounds in marine biology, oceanography, sociology, and anthropology, all affiliated with academic institutions in southern Chile and based in the Los Lagos region, with professional and research experience in coastal and rural communities of the study area. The team acknowledges a shared interest in advocating for more participatory and culturally informed coastal governance, which shaped the interpretive lens applied throughout the study.

Participants were approached through regional fisher federations’ representatives, local fisher organizations’ leaders, and/or municipality fishing departments. After preliminary informative meetings with these “gate-keepers”—through which participants were explicitly informed of the researchers’ institutional affiliations, the academic objectives of the study, and the intended use of the data—workshops were coordinated with their collaboration. No prior personal relationship existed between the research team and the individual participants. Voluntary participants from the community were convened to take part in these activities, resulting in an unintentional, non-systematic and random sample of 64 individuals, including 37 men and 27 women, who were fishers, divers, gleaners and/or housewives integrating 13 fisher, indigenous and/or other local organizations. Given the participatory nature of the workshops, detailed demographic information was not systematically collected for all participants. Consequently, the analysis focuses on the diversity of narratives and voices of the people of the sea in highly vulnerable coastal communities in southern Chile rather than on comparisons among specific participant groups.

**Figure 4:**
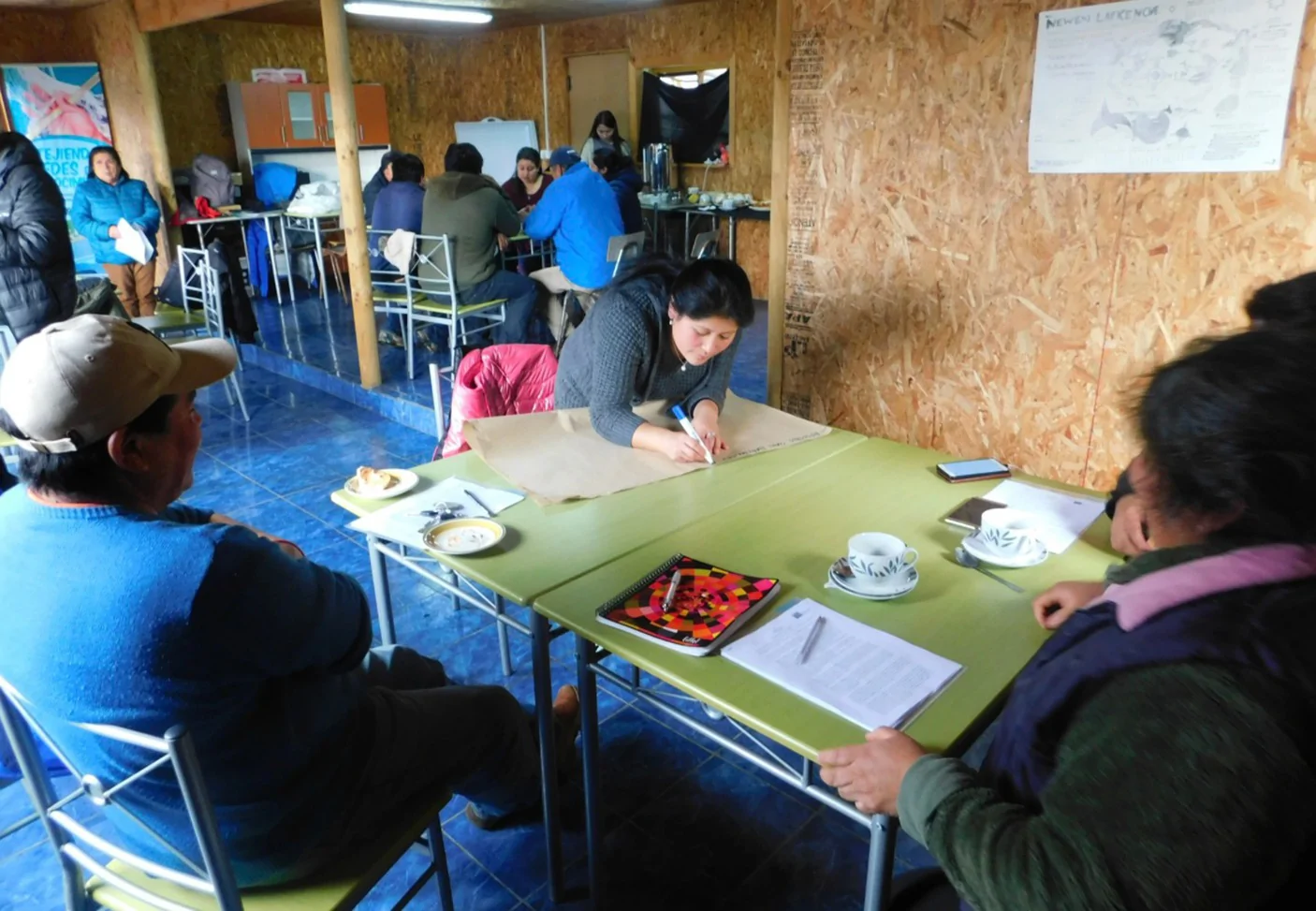
Letter-writing workshop conducted in Mississippi on July 6, 2018. Written informed consent from the identifiable participant in the foreground is available upon request.

The design of the workshops included two main parts. In the first part, the purpose was to gather informants’ direct experiences and lessons from the 2016 HAB event. Participants were invited to form three discussion groups of their own choosing, without any researcher-imposed criteria, and to reflect collectively on their experiences of the crisis. Each group was tasked with drafting an open-ended letter addressed to one specifically assigned recipient, guided by three broad elicitation questions: How did you live through the HAB crisis? What did you learn from it? And what key messages would you like to communicate to your assigned recipient? These open-ended prompts were designed to minimize researcher-imposed framing while encouraging participants to draw on personal and collective memory, articulate the impacts on their livelihoods and communities, and give voice to the knowledge and concerns they considered most relevant to share (Figure 4; for more detail see Supplementary Table S2).

The selection of recipients was informed by the sociological concept of SC (67) and its three-level operational typology. In relation to bonding SC, participants were asked to write letters addressed to their grandchildren. The expectation was to collect information about intergenerational transmission of knowledge, values, and lessons learned from the HAB crisis. With respect to bridging SC, participants wrote letters addressed to members of another coastal community without HAB experience, such as fishing communities in the Biobío region (55). These letters were expected to include shared experiences, warnings, and practical recommendations derived from participants’ experiences.

Finally, in relation to linking SC, participants wrote letters addressed to state authorities and scientists. These letters were intended to elicit expectations, criticisms, and demands regarding environmental monitoring, coastal governance, and crisis communication.

**Figure 5:**
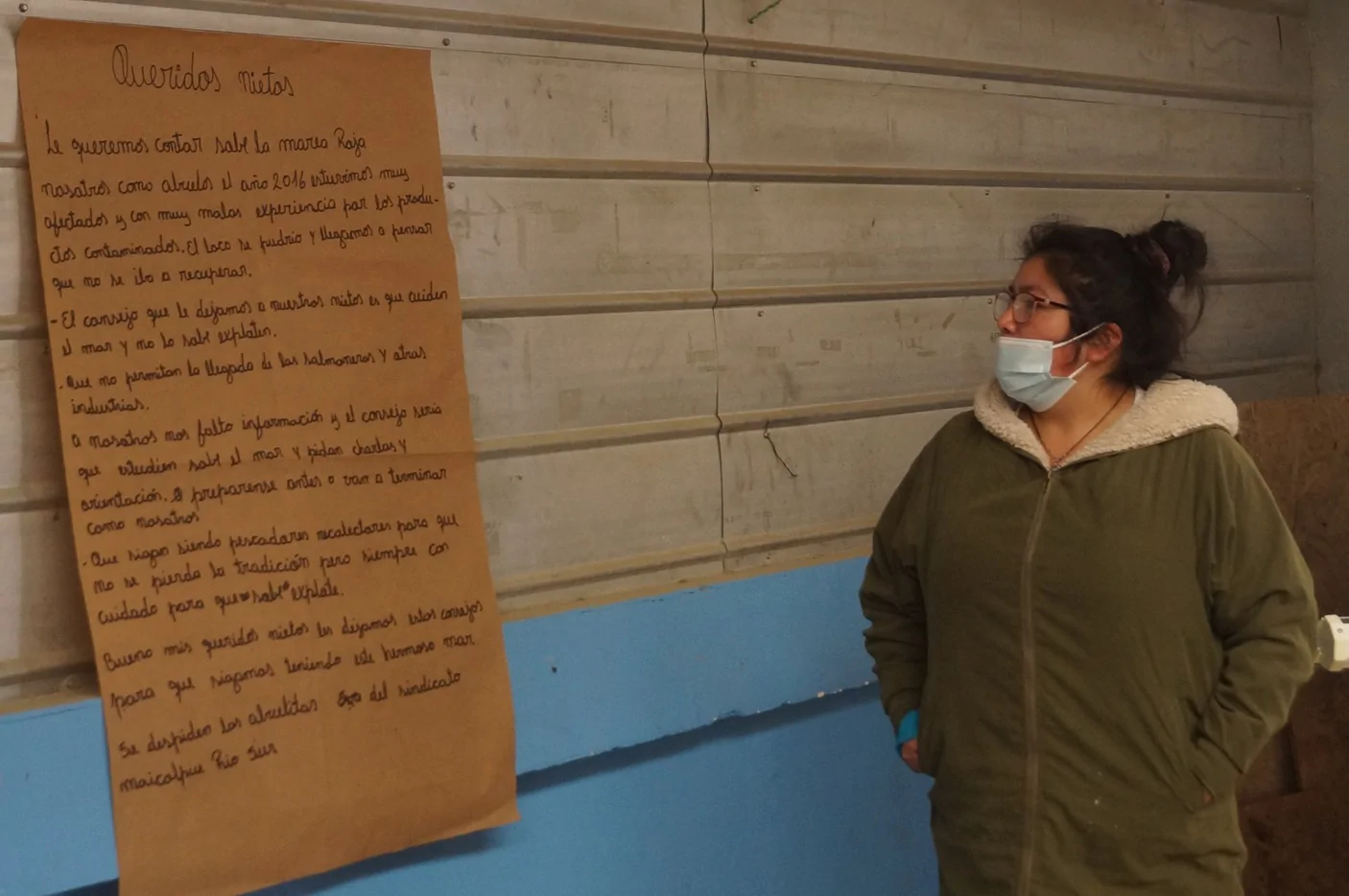
Participant presenting the letter to grandchildren prepared by her discussion group during the workshop in Bahía Mansa (September 28, 2021).

Each group discussion was facilitated by members of the interdisciplinary research team, with the aim of encouraging open discussion and reducing potential barriers related, for instance, with leadership roles. Participants were encouraged to designate a member of the group to act as scribe, although facilitators were available to assist with writing upon request or when literacy and writing skills were limited. Final draft letters were transcribed onto flipcharts, displayed on the walls, and subsequently presented by group representatives, who received comments and feedback from the wider audience (Figure 5).

The second part of the workshop consisted of a presentation on the current state of scientific knowledge about HAB events in Chile. This presentation—prepared by one of the authors—summarized key aspects of HAB evolution, current conditions, and emerging trends in Chile. The purpose was to inform and update the participants about HAB, to further address their concerns and to raise their awareness of associated risks. Fieldnotes were systematically taken by research team members. Each workshop lasted between 1 and 2 h.

The resulting corpus of letters constituted the primary dataset for this study. These were digitized and managed in NVivo qualitative analysis software, which enabled systematic coding and comparison across cases. Initial coding was conducted by one researcher and progressively refined through iterative discussion and validation with the broader research team.

The analysis followed an iterative procedure inspired by grounded theory approaches (68). Open coding first produced a set of inductive codes grounded in participants’ own language and concerns—such as *caja negra* (black box) or *reinventarse para quedarse* (reinvent yourself to stay)—capturing the texture of local experience. Through axial coding, these were organized into broader analytical categories structured around three dimensions: (1) crisis experience and impacts (*consequences*, *uncertainty*, *evidence*, *origin of the problem*); (2) relational resources and strategies (*organization*, *education*, *maintaining tradition*, *new livelihoods*, *caring for the territory*); and (3) institutional dynamics (*information*, *transparency*, *coordination*, *manipulation*, *expectation*). Selective coding then connected these categories to the study’s conceptual framework of SC and community resilience. In parallel, attention was paid to how narratives differed according to the intended recipients of the letters representing the three types of SC. Preliminary findings were informally shared with community representatives in subsequent remote interactions during COVID-19 quarantines to validate interpretations and gather additional feedback.

Five major thematic categories emerged through this process: (1) Impacts and effects of HAB events on livelihoods and well-being; (2) Uncertainty surrounding the causes and dynamics of the phenomenon; (3) Local diagnoses and environmental interpretations; (4) Recommendations and adaptive strategies for future crises; and (5) Expectations and demands directed toward institutional actors.

## 4 Results

The results were structured in two parts. First, we report participants’ descriptions of the direct experiences and impacts associated with HAB events. And then, we present the narratives associated with bonding, bridging and linking SC.

### 4.1 HAB: experiences and impacts

How are the impacts and consequences of the 2016 HAB crisis represented in peoples’ narratives? (RQ1). Across the letters, participants described the HAB crisis as a sudden and disruptive event that affected coastal livelihoods and everyday life. Narratives frequently referred to the closure of fisheries, which interrupted the extraction and commercialization of key benthic resources for extended periods. Participants also recalled the uncertainty that accompanied the appearance of the phenomenon and the difficulties associated with anticipating its duration and consequences within their communities. As one group explained:

“Here nobody expected a red tide. It caught us unprepared and we fell into desperation. We did not know how to move forward or how long it would last. The uncertainty was enormous.” *(letter to fellow fishers, Carelmapu)*

References to economic impacts extended beyond fishing activities themselves. Some groups associated the interruption of extraction with disruptions in processing activities and other forms of employment connected to the fisheries sector. In Carelmapu, for example, narratives referred to the “piure” fishery (i.e., edible tunicate) and to shelling activities traditionally carried out by women. Across several communities, participants linked fishing closures to broader effects on household economies, local commerce, and tourism activities associated with seafood production and consumption.

“It had a strong impact on the whole chain of extraction and processing. The whole chain was cut, in the extraction and the shellfish cleaning, and that affected many women with children.” *(letter to fellow fishers, Carelmapu)*

“In addition, locals and tourists are afraid to eat our seafood because of the possibility of red tide.” *(letter to fellow fishers, Huiro)*

Participants also referred to social and emotional consequences associated with the crisis. In several letters, prolonged fishing closures (particularly in Pasaje Amortajado and Huiro) were described in terms of emotional distress, uncertainty, frustration, and disruptions to established ways of life.

“Many of us had to dedicate ourselves to other activities that were not part of our lifestyle… it caused something like a collective depression, because our way of life changed suddenly.” *(letter to grandchildren, Pasaje Amortajado)*

In addition to economic and emotional impacts, participants addressed changes in everyday food practices and self-consumption fishing. Narratives mentioned restrictions on the consumption and harvesting of seafood during closure periods, as well as the need to rely on alternative food sources.

“We were used to feeding ourselves with shellfish, and from one moment to the next we had to eat things like meat, chicken, lots of potatoes.” *(letter to grandchildren, Amortajado)*

“The sea has always been our work and our food, but during the red tide we realized how fragile everything can be when the sea suddenly closes for us.” *(letter to grandchildren, Mississippi)*

Another recurrent theme across communities concerned questions about why the HAB event had occurred, how long it would last, and what its consequences would be. In some letters, observations of environmental changes were accompanied by discussions of possible contributing factors, including references to human activities and broader transformations in the marine environment.

“First we thought the salmon farms had contaminated us with the dumping 10 miles from here… the water looked dark, brown, oily, and it smelled strong.” *(letter to fellow fishers, Carelmapu)*

“We believe that the occurrence of red tide is linked to potential sources of marine pollution resulting from the discharge of waste and debris by unscrupulous companies.” *(letter to fellow fishers, Mississippi)*

Participants also mobilized environmental observations when describing the emergence and progression of the phenomenon. Changes in water color, unusual residues, the presence or absence of particular species, and other ecological signals were mentioned across several discussion groups. These observations frequently appeared alongside discussions about changing marine conditions and efforts to understand the event through local experience. As one group in Huiro explained:

“The sea began to change color and we started seeing things we had not seen before. The water looked strange, and we knew something was happening even before we heard the word red tide.” *(letter to grandchildren, Huiro)*

Beyond describing ecological signals, a participant group reflected also on the possibility that HABs might become a recurring or enduring feature of coastal life. In these accounts, the phenomenon was not portrayed as a temporary disturbance but as a challenge that communities would need to confront over the long term.

“Scientists say the red tide is here to stay. The state is addressing the issue, because it seems to be serious (…) We’re worried, above all, about what will happen to us as artisanal fishers” *(letter to authorities and scientists, Huiro)*

References to possible industrial or anthropogenic causes of HABs appeared in some letters. At the same time, participants repeatedly referred to concerns about human health and the risks associated with consuming contaminated shellfish. In several communities, these concerns were described in relation to personal experiences, observations of animal mortality, and uncertainty regarding the presence of toxins in the marine environment.

“We want you to know that the Red Tide is real. It is not an invention (…) We even saw it in the saddest way with the experience of what happened to the kitten [which died from eating intoxicated shellfish], which confirmed for us that the poison was there.” *(letter to grandchildren, Carelmapu)*

In communities experiencing these events for the first time, participants referred to limited knowledge regarding toxin symptoms and potential health effects. Several letters expressed concerns about poisoning risks, uncertainty surrounding monitoring procedures, and the implications of HAB events for seafood safety and consumer confidence.

“We do not have knowledge about the symptoms produced by the red tide… we know that the red tide can gravely affect (…) the health of our families by the consumption of contaminated shellfish.” *(letter to fellow fishers, Mississippi)*

“Any error or inadequate handling of the process to determine whether there is a red tide or not is going to cost us our jobs, our food, and our own health and that of the population.” *(letter to authorities and scientists, Huiro)*

Although narratives varied among communities, recurring themes emerged in all five cases, as summarized in Table 1. Participants described economic disruption affecting not only fishing activities but the broader productive chain and household economies. References to social and emotional consequences, changes in food practices, and local interpretations of environmental change also appeared throughout the letters, particularly when participants addressed the emergence and progression of the phenomenon within their communities.

**Table 1:** Experiences and impacts of the 2016 HAB across coastal communities.

Dimension	Key narratives
Economic impacts	Fishing closures interrupted extraction and commercialization of benthic resources; processing activities and women’s employment were disrupted; local commerce and tourism declined
Productive chain disruption	Effects extended beyond fisheries to shellfish cleaning, processing, and household economies
Social well-being	Prolonged closures generated anxiety, frustration, and collective distress; established ways of life were suddenly altered
Food practices	Restrictions on seafood harvesting and self-consumption led communities to rely on alternative food sources
Environmental interpretations	Changes in water color, unusual residues, and species decline were mobilized as local indicators of the event’s emergence and progression

### 4.2 Social capitals: bonding, bridging, linking

How do participants narrate and attribute significance to social relationships associated with bonding, bridging, and linking SC in relation to the HAB crisis? (RQ2). The letters addressed to grandchildren, other fishing communities, and institutional authorities and scientists contained recurring references to family ties, community relationships, inter-community exchanges, and interactions with external actors. These narratives provide insight about the social relationships and institutional connections that participants considered important when reflecting on past experiences and future environmental challenges.

#### 4.2.1 Bonding social capital

Letters addressed to grandchildren frequently referred to family relationships, shared experiences, and connections to places. Across communities, and due to the elicitation technique, participants used these letters to communicate advice, concerns, memories, and expectations regarding the future of fishing livelihoods and coastal territories.

Participants offered advice regarding the care of marine ecosystems and the continuity of fishing practices. Across several letters, the sea was discussed not only as a source of livelihood but also as an element closely associated with family histories, community life, and future generations.

“Take care of the sea and do not overexploit it… continue being fishers and gatherers so that the tradition is not lost.” *(letter to grandchildren, Bahía Mansa)*

At the same time, some groups encouraged younger generations to pursue educational opportunities, develop alternative skills, and consider additional livelihood options. References to diversification often appeared alongside reflections on environmental uncertainty and the possibility of future disruptions affecting fisheries and coastal livelihoods.

“If the red tide should arrive, we advise you to reinvent yourselves and take advantage of other local natural resources, so you will not have to leave and can remain in your territories.”*(letter to grandchildren, Mississippi)*

“We want to give you some advice for your life: study hard to have another profession. In this world, you have to learn to ‘put your eggs in different baskets’ so that, when the sea is closed… you have the freedom to decide.” *(letter to grandchildren, Carelmapu)*

Letters included lessons learned during the HAB crisis and expressed a desire to share these experiences with younger generations. Participants treated the letters as opportunities to communicate advice, warnings, and reflections about future environmental events. In some communities, these messages were also accompanied by references to indigenous identity, language, and relationships with the sea.

“We want to tell you this story so you are better prepared than we were and do not have to live the same thing.” *(letter to grandchildren, Pasaje Amortajado)*

“That is why we ask you to be respectful, and also not to be ashamed of speaking your language, Mapudungun; as you know, it is your mother-tongue, and our sea is our ‘Lafken’ in Mapudungun.” *(letter to grandchildren, Mississippi)*

Across the five communities, letters addressed to grandchildren frequently combined memories of the HAB crisis with advice concerning future livelihoods, environmental stewardship, and community life. References to family relationships, shared experiences, cultural traditions, and intergenerational communication appeared throughout these narratives.

#### 4.2.2 Bridging social capital

Letters addressed to other fishing communities included references to shared experiences, mutual learning, and exchanges among coastal communities facing similar environmental challenges. Participants used these letters to communicate lessons from the 2016 HAB crisis, offer advice, and reflect on the challenges of responding to environmental uncertainty in fishing-dependent territories.

“In the face of the threat of red tide we recommend: pay attention to the signals of the sea — temperature, color, smell and the species.” *(letter to fellow fishers, Carelmapu)*

Across several communities, participants referred to the value of exchanging experiences with others who depend on marine resources and face similar environmental risks. Some letters contained recommendations regarding preparedness, diversification, environmental stewardship, and collective organization. Rather than focusing exclusively on local circumstances, participants often framed the HAB crisis as a challenge shared by coastal communities more broadly.

For example, participants from Mississippi wrote:

“We want to share what happened to us so that other fishing communities know what can occur and can be prepared when the sea shows these signs.” *(letter to fellow fishers, Mississippi)*

Likewise, participants from Huiro stressed the importance of coordinated preparedness across coastal territories:

“What happened here should serve as a lesson for other communities, because these events may happen again and we must be prepared.” *(letter to fellow fishers, Huiro)*

Taken together, the letters addressed to other fishing communities emphasized the exchange of experiences, practical advice, and reflections derived from the HAB crisis. References to cooperation, shared challenges, and mutual learning appeared across communities, suggesting that participants often interpreted their experiences in relation to broader networks of coastal communities facing environmental uncertainty.

Furthermore, letters addressed to other fishing communities referred to collective organization and unity. Some communities associated organization with efforts to seek information, request accountability, and demand greater transparency regarding marine pollution and environmental management, as illustrated in the following quotations:

“We advise you to stay informed through your organizations, so as to better understand how the red tide could affect your localities.” *(letter to fellow fishers, Mississippi)*

“It is important to organize yourselves to request information and support to face this situation and to demand greater oversight of other factors that are polluting our sea.” *(letter to fellow fishers, Huiro)*

Other letters referred to organization as an important means of engaging with external actors and accessing support beyond the community. Participants associated collective organization with opportunities to communicate community concerns, seek assistance, and interact with institutions and other organizations, as conveyed by a women’s group in Bahía Mansa:

“Stay organized and united to help each other (…) [and to] manage aid with universities, deputies, and federations.” *(letter to fellow fishers, Bahía Mansa)*

Finally, letters connected collective organization with concerns about future HAB events and the protection of coastal livelihoods and community interests, as expressed in the following excerpt:

“Stay united with other fishers to defend your rights. Pollution or red tide can recur and you have to be prepared.” *(letter to fellow fishers, Pasaje Amortajado)*

Taken together, the letters addressed to other fishing communities frequently drew on the experience of the 2016 HAB crisis to communicate advice, concerns, and lessons for the future. Across communities, participants repeatedly referred to the importance of maintaining connections with others, sharing information, and organizing collectively when facing environmental uncertainty. References to participation, access to information, representation, and preparedness for future events appeared throughout these narratives.

#### 4.2.3 Linking social capital

Letters addressed to authorities and scientists contained frequent references to relationships between fishing communities and the institutional actors involved in environmental management, scientific research, and HAB monitoring. Participants often reflected on the role of public institutions and technical experts in responding to environmental crises and communicating information to coastal communities.

Participants expressed concerns regarding transparency in HAB monitoring systems and the limited flow of information between scientists, authorities, and fishing communities. Across several letters, participants referred to difficulties accessing timely information about the causes, evolution, and implications of the HAB event.

“We ask the authorities to improve communication channels so we can know more about red tide and act in time when it affects our livelihoods.” *(letter to authorities and scientists, Bahía Mansa)*

Participants also addressed scientists directly, emphasizing the importance of communicating research findings in accessible ways and sharing information with coastal communities. Most letters expressed interest in greater interaction between researchers and fishers, particularly regarding environmental monitoring and the interpretation of ecological changes.

“To the scientists we ask that they share their findings about red tide and its prevention so that the whole community understands how dangerous it can be.” *(letter to authorities and scientists, Bahía Mansa)*

References to HAB monitoring programs often appeared alongside broader reflections on information, communication, and trust. Some groups commented on the availability, clarity, and accessibility of information related to environmental risks and HAB management.

Letters addressed to authorities contained demands, questions about institutional responses, and expectations regarding the responsibilities of public actors during environmental crises.

“We call for immediate action. Do not wait for desperation to force us again to take over the highways in order for you to finally recognize our demands.” *(letter to authorities and scientists, Carelmapu)*

“We demand that you come to the caleta to give us information and clear our doubts… What proposals and solutions exist from the government?” *(letter to authorities and scientists, Mississippi)*

Some letters also referred to the capacity of fishing communities to organize collectively and make their concerns visible in public and political arenas. These messages frequently combined references to rights, representation, participation, and collective mobilization when discussing future responses to HAB events and other environmental challenges.

In general, when addressing institutional actors, the narratives focused on communication, information sharing, institutional accountability, participation, and community representation. Across the five communities, participants repeatedly reflected on their relationships with external actors and institutions when discussing both past experiences of the 2016 HAB crisis and future environmental uncertainties.

Table 2 presents a summary of the narrative functions and outcomes emerged from the data at each SC level. Next, based on the results presented, we discuss the implications of our findings for understanding and supporting community resilience and adaptive capacity, and for informing future HAB governance (RQ3).

**Table 2:** Social capital narratives revealed through the letters.

Social capital dimension	Letter recipient	Narrative functions	Narrative outcome
Bonding	Grandchildren	Transmission of knowledge and identity; advice on livelihood diversification; intergenerational warnings about future environmental events	Reinforces cultural continuity and community preparedness
Bridging	Other fishing communities	Sharing experiences and lessons from the crisis; recommendations for preparedness and stewardship; promotion of collective organization	Facilitates horizontal learning and inter-community solidarity
Linking	Authorities and scientists	Demands for transparency and timely information; requests for accessible scientific communication; pressure for institutional accountability	Reveals governance tensions and expectations toward external actors

## 5 Discussion

### 5.1 HABs as socio-environmental crises interpreted through experience and local ecological knowledge

The letters portray the 2016 HAB event as far more than an ecological disturbance or a temporary interruption of fishing activities. Participants consistently described the crisis as a multidimensional experience affecting livelihoods, food practices, family well-being, and everyday relationships with coastal environments. These accounts reinforce the view that HABs are not merely sanitary or biophysical events, but socio-environmental crises whose consequences unfold through the social and cultural fabric of coastal communities (13, 49). While previous research has primarily focused on biological monitoring, health risk mitigation, and economic cost quantification (47, 48), the letters analyzed here highlight how HABs are experienced, interpreted, and remembered through everyday life, social relationships, and territorial identities.

A particularly relevant finding concerns the role of experiential and locally grounded knowledge in making sense of environmental change. Accounts frequently combine observations of unusual environmental conditions—such as changes in water color, texture, smell, and marine species behavior—with their own explanations regarding the evolution and consequences of the bloom. These narratives reflect processes of environmental sense-making that extend beyond formal scientific monitoring and draw upon long-term familiarity with coastal ecosystems. This observation is consistent with Berkes (28), who argues that LEK provides important frameworks through which communities interpret environmental variability and recognize anomalous ecological signals, even when facing novel or unprecedented disturbances. This is illustrated in the letters from Huiro, where participants described recognizing environmental anomalies—changes in water color, unusual residues, unfamiliar smells—before the event was officially identified: *“we knew something was happening even before we heard the word red tide”* (letter to grandchildren, Huiro). Such accounts suggest that LEK functioned as an early warning system operating in parallel with, rather than in opposition to, formal monitoring (28).

The findings also provide an interesting contrast to previous studies of the 2016 crisis in the Chiloé archipelago. In areas where HAB events have been documented for decades, research has emphasized epistemic disputes regarding the industrial or natural origins of the bloom (18, 19). In the rural villages examined here, however, participants appeared less concerned with establishing definitive causal explanations and more focused on understanding the consequences of the event for their livelihoods, food security, and family well-being. While industrial pollution remained a source of suspicion, the dominant concern across the letters was how to confront and navigate the disruptions generated by the crisis.

These reflections situate HABs within broader socio-environmental contexts where environmental disruption, economic vulnerability, and cultural meanings become deeply intertwined. This interpretation aligns with resilience perspectives emphasizing learning, knowledge, and sense-making as important components of responses to environmental change. At a broader level, the findings point to the cultural dimensions of resilience, whereby experiences, interpretations, and shared meanings influence how people anticipate and navigate environmental uncertainty (30, 31).

### 5.2 Social capital as a cultural and relational lens

A central contribution of this study lies in its use of SC as an interpretive framework. Unlike approaches that seek to measure this concept as a predictor of recovery outcomes or resilience performance (40, 69), this study uses bonding, bridging, and linking SC as analytical lenses for examining how participants assign meaning to the HAB crisis. The findings suggest that experiences of environmental disruption are rarely recalled in isolation; rather, they are remembered and narrated through relationships with family members, neighbors, fishing organizations, other communities, scientists, and public institutions.

The narratives associated with bonding SC frequently emphasized family support, solidarity, intergenerational continuity, and territorial identity. The letters often linked close social relationships to both material forms of support and the transmission of intangible resources, including traditions, local knowledge, and cultural values. Compared with studies reporting social fragmentation and declining trust following the 2016 crisis in more urbanized settings (19, 21), the letters analyzed here placed greater emphasis on continuity, mutual support, and the maintenance of collective identity. The narratives highlight the importance that informants themselves attribute to close social ties when reflecting on past experiences and future uncertainties. This is evident in letters such as the one from Mississippi, where participants explicitly connected cultural identity, language, and territorial belonging: *“not to be ashamed of speaking your language, Mapudungun; as you know, it is your mother-tongue, and our sea is our ‘Lafken’“*(letter to grandchildren, Mississippi). The sea appears here not only as a livelihood resource but also as a cultural anchor associated with the transmission of identity, memory, and continuity across generations.

Bridging SC emerged through letters addressed to other fishing communities. These accounts highlighted the value of sharing experiences, warnings, and environmental observations beyond local boundaries. Participants frequently framed the lessons learned from the HAB crisis as knowledge that should be communicated to others facing similar risks. In this sense, bridging relationships are portrayed as channels through which experiential knowledge, practical lessons, and environmental signals can circulate among communities. Importantly, these exchanges were presented not merely as responses to a specific event, but as contributions to broader processes of collective learning in contexts of environmental uncertainty. Participants from Huiro made this explicit: *“what happened here should serve as a lesson for other communities, because these events may happen again and we must be prepared”* (letter to fellow fishers, Huiro). This framing positions experiential knowledge as a place-based resource whose value extends beyond local contexts through processes of knowledge exchange and social learning.

The narratives associated with linking SC reveal more complex and ambivalent relationships. Participants frequently expressed frustration regarding perceived deficiencies in communication, transparency, and institutional responsiveness. At the same time, however, the letters rarely advocated disengagement. Instead, they repeatedly called for greater dialogue, accountability, participation, and information sharing. This finding contrasts with studies describing a near-complete breakdown of trust between communities and institutions following the 2016 crisis (21). Although institutional legitimacy was often questioned, the reflections continued to portray relationships with authorities and scientists as important and potentially necessary. In this sense, the “social pipelines” connecting communities to institutional actors remained active sites of negotiation, expectation, and community agency.

These findings support critiques suggesting that the value of social capital is context-dependent and should not be understood as a one-size-fits-all solution to environmental challenges (42, 46). The letters reveal both enabling and constraining dimensions of social relationships, including solidarity and support, but also frustration, exclusion, and unequal access to decision-making. By focusing on the content and meaning of these relationships, the study shows how social ties become part of the interpretive processes through which individuals construct understandings of environmental change and imagine future possibilities for action.

Taken together, the three dimensions of SC illuminate relational aspects of the HAB crisis that are often difficult to capture through conventional monitoring and impact assessments. Across the letters, informants repeatedly reflected on issues of support, cooperation, communication, trust, knowledge sharing, and institutional engagement. These dimensions appear throughout the letters as important elements through which informants interpret risks, discuss responses to environmental disruptions, and reflect on their relationships with governance systems. Viewed in this way, this perspective serves less as an explanatory variable than as a cultural and relational lens through which the social foundations of potential adaptive responses become visible (33, 34, 44).

### 5.3 Collective memory, future-oriented narratives, and adaptive capacity

One of the most distinctive aspects of the findings is their strong prospective orientation. Although accounts refer to an event that occurred several years earlier, the letters rarely functioned as simple recollections of the past. Instead, they became vehicles for communicating lessons, warnings, concerns, and recommendations to future generations, fellow fishers, and other coastal communities. The epistolary format proved particularly valuable in this regard, encouraging participants not only to remember the crisis but also to project its practical and moral significance into the future. In this sense, the letters linked memories of the crisis to reflections about future action.

This future-oriented dimension is particularly relevant when considering adaptive capacity, broadly understood as the ability to anticipate, learn from, and adjust to environmental change (30, 35). The letters provide insight into how coastal dwellers envision future capacities, needs, and responses to environmental change, contributing to broader discussions of adaptive capacity. Expressions encouraging younger generations to diversify livelihoods, pursue education, or avoid excessive dependence on a single activity reveal concerns about future vulnerability and aspirations for greater flexibility in the face of environmental uncertainty. These reflections indicate that participants frequently associated adaptation more with diversification, learning, and greater flexibility in anticipation of future disruptions, rather bouncing back to pre-crisis conditions (31, 43, 70). This is particularly evident in letters from Huiro and Mississippi, where participants advised future generations that *“the red tide is here to stay”* and to *“reinvent yourselves and take advantage of other local natural resources.”* These formulations suggest that participants associated adaptive capacity less with restoring a prior state than with cultivating flexibility and diversification as enduring orientations toward environmental uncertainty.

The letters also suggest that collective memory may contribute to future preparedness. Individuals who experienced the bloom frequently transformed their experiences into warnings, lessons, and practical advice for others. Ecological observations associated with the 2016 bloom—such as changes in water color, smell, or marine behavior—were often presented as signals that future generations should learn to recognize. Consistent with Berkes (28), these findings suggest that LEK can function not only as a way of interpreting past events but also as a means of anticipating and making sense of future environmental change.

Ultimately, the letters highlight dimensions of preparedness and adaptation that extend beyond material resources or observable responses. It also appears in the ways communities imagine, discuss, and communicate possible futures. People’s stories portray adaptive capacity as a set of aspirations, concerns, lessons, and anticipated courses of action that participants associated with preparing for future HAB events. In this sense, the letters provide access to dimensions of preparedness and adaptation that are often overlooked in more conventional assessments of environmental crises.

### 5.4 Bridging, linking, and environmental governance

The letters addressed to other fishing communities, scientists, and authorities illustrate that participants frequently interpreted and discussed the HAB crisis through relationships connecting communities with actors operating beyond the local scale. The narratives suggest that bridging and linking relationships are valued not only for access to resources but also for their potential to facilitate communication, learning, representation, and participation.

The letters written to other fishing communities frequently emphasized the importance of sharing experiences and environmental observations. Accounts portrayed these exchanges as opportunities for mutual learning and collective preparedness. Unlike studies emphasizing political mobilization, conflict and fragmentation as primary expressions of inter-community relationships during the 2016 crisis (19), the narratives analyzed here highlighted more collaborative forms of interaction. The epistolary circulation of environmental warnings, practical advice, and experiential knowledge suggests that participants regarded communication among communities as an important element of anticipating and responding to future environmental challenges.

The findings also suggest that several dimensions frequently identified in the HAB governance literature—including trust, communication, stakeholder participation, and the integration of local knowledge—are fundamentally relational processes (22, 27). This study approached these dimensions through participants’ accounts, providing insight into how they are experienced, interpreted, and evaluated by coastal residents. The letters reveal how coastal residents themselves experienced communication processes, evaluated institutional responsiveness, shared environmental observations, and reflected on opportunities for collective action. Thereby, they provide insight into social dimensions of governance that are often overlooked by technically oriented monitoring and management frameworks.

More broadly, the findings help illuminate dimensions of HAB governance that remain difficult to capture through formal and institutional monitoring systems. While monitoring programs generate critical information about environmental and public health risks, the letters reveal how issues of communication, trust, participation, and knowledge exchange are experienced and interpreted by coastal residents themselves. Examining these relational dimensions through a SC lens therefore contributes to ongoing efforts to develop more legitimate, participatory, and adaptive approaches to HAB governance (18, 25).

The narratives directed toward authorities and scientists revealed persistent tensions regarding information flows, environmental monitoring, and institutional transparency. Nevertheless, participating individuals rarely rejected scientific knowledge or governance institutions outright. Instead, they repeatedly expressed a desire for more accessible information, clearer communication, and more meaningful opportunities for participation. This distinction is important because trust in environmental governance depends not only on scientific accuracy but also on processes of communication, engagement, and legitimacy (34). The letters from Bahía Mansa illustrate this clearly: *“we ask the authorities to improve communication channels so we can know more about red tide and act in time when it affects our livelihoods”* (letter to authorities and scientists, Bahía Mansa). This statement reveals that institutional legitimacy is evaluated not only in terms of technical accuracy but also in terms of accessibility, timeliness, and the perceived responsiveness of governance systems to everyday livelihood concerns.

These findings suggest that communities value not only access to information but also participation in its interpretation and discussion. The letters indicate that participants placed considerable value on opportunities for dialogue, knowledge sharing, and participation. From this perspective, HAB governance may benefit from complementing technical monitoring systems with more relational forms of engagement that recognize local experiences, concerns, and knowledge systems. Such approaches are increasingly recognized as important for strengthening the legitimacy, credibility, and social relevance of environmental governance (22, 25).

### 5.5 Implications for resilience and HAB governance

Taken together, our findings contribute to ongoing discussions regarding community resilience and adaptive capacity in coastal social-ecological systems. Rather than treating resilience as a stable community attribute, contemporary resilience perspectives increasingly emphasize its relational and processual dimensions. The narratives examined here are consistent with this view, portraying resilience as a culturally embedded process through which people interpret environmental change, mobilize social relationships, and imagine future possibilities for action. This perspective aligns with approaches that emphasize the active articulation of multiple resources—including knowledge, leadership, institutions, and social relationships—in contexts of uncertainty and change (30, 31). Without attempting to measure or demonstrate the effects of SC on resilience outcomes, this framework helps to illuminate the social resources, expectations, and tensions through which coastal residents interpret environmental crises and envision future capacities for response.

For policymakers, these findings highlight the limitations of predominantly top-down approaches to HAB governance. Many of the concerns expressed in the letters relate less to technical monitoring than to communication, participation, and trust. While HAB management is commonly evaluated in terms of monitoring capacity, regulatory performance, and public health outcomes—for example, toxin analyses, enforcement activities, and poisoning rates—participants’ letters reveal how coastal residents experience communication processes, assess institutional responsiveness, and reflect on opportunities for participation. These concerns are particularly relevant in a governance context characterized by fragmented institutional responsibilities and largely reactive management efforts (18). Therefore, strengthening communication channels, supporting horizontal learning among fishing communities, and establishing platforms for dialogue and social learning between multiple actors may represent important complements to existing monitoring and management systems.

More broadly, the study demonstrates the value of narrative approaches for understanding dimensions of environmental governance that are often difficult to capture through technical, biophysical and economic assessments. Evidence on how environmental crises are remembered, interpreted, and projected into the future, the findings shed lights on how affected communities navigate the relationships between local realities and institutional frameworks during periods of environmental disruption. By highlighting local experiences, relational dynamics, and future-oriented reflections, the study highlights opportunities for developing HAB governance approaches that place greater emphasis on communication, participation, knowledge exchange, and institutional legitimacy.

The narrative and retrospective nature of this study opens questions that extend beyond the scope of the present research. Because the letters were produced several years after the event, they capture enduring interpretations and lessons emerging from participants’ longer-term reflections on the crisis. Similarly, the study focuses on how participants describe, value, and imagine relational resources, rather than on the direct observation of collective action, resource mobilization, or adaptive responses. Furthermore, as no HAB event of comparable magnitude has occurred in the study area since 2016, it remains unclear how the lessons, warnings, and aspirations articulated in these narratives might influence responses to future environmental disturbances. These considerations point toward fruitful avenues for future research, including longitudinal studies of environmental crises, comparative analyses across coastal communities, examinations of how relational resources are translated into collective practices, and mixed-methods approaches exploring the connections between SC narratives, adaptive actions, and resilience processes over time.

## 6 Conclusion

This study examined how residents of five rural fishing villages in southern Chile retrospectively narrated the 2016 HAB crisis through a participatory epistolary exercise analyzed using a cultural approach to social capital. The letters revealed how participants interpreted and remembered the crisis through everyday experiences, LEK, and relationships with family members, neighbors, other fishing communities, authorities, and scientists. In doing so, they highlighted relational and cultural dimensions commonly associated with resilience perspectives, including the interpretation of environmental change, the mobilization of social relationships, and reflection on future possibilities for action.

A central contribution of the study is conceptual and methodological. Rather than measuring social capital or assessing its effects on resilience outcomes, bonding, bridging, and linking ties were used as interpretive lenses for understanding how participants framed their experiences and concerns. The elicited epistolary approach proved particularly valuable for capturing both retrospective accounts and future-oriented reflections, revealing recurring themes of family solidarity, intergenerational knowledge transmission, collaboration among fishing communities, and demands for greater institutional transparency. These findings suggest that environmental crises are understood not only through their tangible impacts but also through the social relationships and institutional interactions that shape people’s experiences of disruption.

The study also highlights the importance of future-oriented narratives. Participants transformed past experiences into lessons, warnings, aspirations, and recommendations directed toward future generations, fellow fishers, scientists, and public authorities. While these narratives do not constitute adaptive capacity in themselves, they illuminate forms of knowledge and relational awareness that participants regarded as relevant for confronting future environmental challenges.

Several implications for HAB governance emerge from these findings. The letters emphasized the need for clearer crisis communication, greater transparency, stronger collaboration between institutions and coastal communities, and recognition of local experiential knowledge. Together, these concerns suggest that effective HAB governance depends not only on technical monitoring and forecasting systems but also on dialogue, trust, and participation.

Ultimately, the experiences and reflections documented in these letters suggest that HAB governance should evolve beyond a predominantly reactive and health-centered model toward preventive, participatory, and adaptive approaches that protect public health while also supporting sustainable livelihoods and the long-term resilience of fishing communities.

## Data Availability

The raw data supporting the conclusions of this article will be made available by the authors, without undue reservation.

## Footnotes

1 Throughout the manuscript, the term harmful algal bloom (HAB) is used as the scientific and analytical reference to the phenomenon. The expression “red tide” (i.e., “marea roja” in Spanish) is retained only in participants’ quotations to preserve the original wording used during the workshops and in the letters.

2 The IFOP-CREAN program has also recently implemented outreach initiatives to disseminate HAB-related information through early-warning products, digital platforms, technical guides, and stakeholder workshops targeting coastal users and institutions.

3 The original fieldwork plan was affected and extended, first due to the social upheaval in Chile (October 2019–March 2020), and then due to the COVID-19 pandemic and the prolonged quarantines (March 2020–September 2022).
