# Correction: Fishing community resilience to harmful algal blooms: social capital narratives of the 2016 crisis in southern Chile

**DOI:** 10.3389/fpubh.2026.1960433

**Published:** 2026-08-18

**Authors:** Frontiers Production Office

**Affiliations:** Frontiers Media SA, Lausanne, Switzerland

**Keywords:** adaptive capacity, environmental governance, crisis communication, local ecological knowledge, participatory research, red tide, small-scale fisheries

The funder ANID-FONDECYT 11171068 to AM was erroneously omitted. The correct funding statement reads:

The author(s) declared that financial support was received for this work and/or its publication. AM was funded by ANID-FONDECYT 11171068. PD was funded by the ANID-FONDECYT 1262020 and by the Centro Interdisciplinario para la Investigación Acuícola - Investigación Aplicada (INCAR2), CIA250009, ANID, Chile, both from the Chilean National Agency for Research and Development (ANID).

## Correction note

This article has been corrected with minor changes. These changes do not impact the scientific content of the article.

The original version of this article has been updated.

