# Supplementary material for: Fishing community resilience to harmful algal blooms: social capital narratives of the 2016 crisis in southern Chile
Source: Front Public Health. 2026 Aug 5;14:1872462. doi: 10.3389/fpubh.2026.1872462 (PMC13543661; doi:10.3389/fpubh.2026.1872462)
Supplement: Supplementary file 1 [file Supplementary_file_1.pdf]

Supplementary Material

1 Study area sociodemographic characterization

There is lack of detailed information at the scale of the fishing villages (caletas) included in this study, namely Mississippi, Huiro, Bahía Mansa, Amortajado and Carelmapu. However, it is possible to estimate some of their conditions based on communal data. Based on PNUD (2022) reports, San José de la Mariquina, Corral, San Juan de la Costa, and Maullín are all classified within the "Low" human development tier in Chile, a category representing communes with HDI scores below 0.549 and characterized by significant structural vulnerability and socio-economic stagnation. Collectively, these communes reflect a reality of deep territorial inequality where high rurality, geographic isolation from regional capitals, and a lack of municipal financial autonomy create persistent barriers to development. A critical highlight is San Juan de la Costa, which holds the national minimum score for both the overall HDI (0.297–0.302) and the health dimension (0.455), illustrating extreme levels of precariousness. An important part of the population in the study area identifies with ethnic groups, particularly Mapuche-Williche. The vulnerability of these communes is further exacerbated by their economic reliance on primary-sector activities like artisanal fishing and agriculture.

Table S1: Communal HDI Scores (PNUD 2004)

Commune	Caleta	HDI Category	HDI Score	Health	Education	Income	Indigenous pop.
San José de la Mariquina	Mississippi	Low	0.516	0.712	0.739	0.262	42.2%
Corral	Huiro	Low	0.507	0.745	0.72	0.243	43.9%
San Juan de la Costa	Bahía Mansa	Low	0.302	0.455	0.617	0.098	81.7%
Maullín	Amortajado & Carelmapu	Low	0.495	0.721	0.638	0.264	15.1%
National Range (Min–Max)		—	0.297 – 0.961	0.455 – 0.994	0.559 – 1.000	0.083 – 1.000	9.2%

Figure legend: HDI Categories and Score Ranges: Very High: From 0.800 to 1; High: From 0.700 to 0.799; Medium-high: From 0.625 to 0.699; Medium-low: From 0.550 to 0.624; Low: From 0 to 0.549. Source: PNUD (2024). El Desarrollo Humano de las Comunas de Chile. Santiago, Chile: Programa de las Naciones Unidas para el Desarrollo. https://www.undp.org/es/chile/publicaciones/el-desarrollo-humano-de-las-comunas-de-chile

2 Sample description

Workshops participant sampling draws on a “gate-keeper” based, unintentional, non-systematic random sample, including coastal community members associated with small-scale fisheries. No exclusion criteria were applied. Due to the voluntary call to participate, additional information about response rates or withdrawing participants is not available. No material compensation or incentives were provided to gatekeepers and participants.

Table S2: Sample description

	Workshops	Participants			Fisher/Local Organizations	Letters
		Men	Women	Subtotal		
Mississippi	1	8	5	13	3	3
Huiro	1	9	6	15	3	3
Bahía Mansa	1	2	10	12	2	3
Amortajado	1	11	3	14	3	3
Carelmapu	1	7	3	10	2	3
Total	1	37	27	64	13	15

3 Workshop data collection procedures

The letter-writing activity was based on the following elicitation instructions or prompts. We are interested in your memories, experiences, reflections, and perspectives. There are no right or wrong ideas. All contributions will be treated as a group product, confidentially and used only for research purposes.

Instructions for participants: “We invite you to recall and reflect about your individual and collective experiences with the 2016 HAB event. And then, write together a letter capturing the ideas and messages about the "red tide" event and its effects on your community. Please address the letter to ____________. There is no correct format and no minimum length. A member our team will participate in the group and assist with the writing”.

Indications for facilitators: In the letters, the first and permanent goal is to allow participates to express themselves spontaneously, and with the lowest intervention of the research team. If (or once)

the spontaneous talk halts and long silence is generated, you can intervene and encourage participants to reflect on questions such as: What happened in your community during the crisis? How did the event affect your family, work, and daily life? What emotions, concerns, or challenges do you remember? Who helped you during this period? How did people in the community respond? What role did organizations, authorities, neighbours, or family networks play? What lessons did you learn from this experience? What would you like others to know about what happened?
